# Effect of Savirin or Ticagrelor Treatment on the Expression of Commonly Used Reference Genes in *Staphylococcus aureus*

**DOI:** 10.3390/microorganisms11020336

**Published:** 2023-01-29

**Authors:** Narayan Pant, Catherine Rush, Jeffrey Warner, Damon P. Eisen

**Affiliations:** 1 College of Medicine and Dentistry, James Cook University, Townsville, QLD 4811, Australia; 2 Australian Institute of Tropical Health and Medicine, Townsville, QLD 4811, Australia

**Keywords:** *S. aureus*, savirin, ticagrelor, reference gene, qRTPCR

## Abstract

Reference genes are frequently used for the normalization of quantitative reverse transcriptase PCR (qRTPCR) data in gene expression studies. *Staphylococcus aureus* is one of the most common causes of biofilm-related infections. Savirin and ticagrelor show in vitro as well as in vivo antibiofilm activity against *S. aureus*. The main aim of this study was to identify the most stably expressed reference genes to study the effect of these molecules on genes in a strong biofilm producing *S. aureus* isolate isolated from biofilm-related infection. Quantitative real-time PCR was performed by using relative quantification method. Four different algorithms, delta Ct, normfinder, bestkeeper, and genorm, followed by a comprehensive analysis was used to identify the most stable reference genes from a list of sixteen different candidate reference genes. All four algorithms reported different results, with some comparable findings among some methods. In the comprehensive analysis of the results of all the algorithms used, the most stable reference genes found were *spa*, *rpoD*, and *pyk* for savirin treatment experiment and *gapdH*, *gyrA*, and *gmk* for ticagrelor treatment experiment. The optimal number of reference genes required was two for both the experimental conditions. Despite having some drawbacks, each algorithm can reliably determine an appropriate reference gene independently. However, based on consensus ranking and the required optimal number of reference genes reported, *spa* and *rpoD* were the most appropriate reference genes for savirin treatment experiment, and *gapdH* and *gyrA* were most appropriate for ticagrelor treatment experiment. This study provides baseline data on reference genes to study the effect of savirin or ticagrelor treatment on the expression of potential reference genes in *S. aureus*. We recommend prior re-validation of reference genes on a case-by-case basis before they can be used.

## 1. Introduction

Quantitative reverse transcriptase PCR (qRTPCR) was first established in 1992; since then, it has been routinely used for the analysis of gene expression [1]. Errors may be introduced during the RNA extraction, reverse transcription and amplification stages of qRTPCR experiment [2]. Therefore, normalization of qRTPCR data to compensate for errors is essential for the generation of reliable results [2]. While microarray analysis provides an opportunity to analyze the expression of several genes simultaneously, the results still need to be validated using qRTPCR [3]. Quantitative reverse transcriptase PCR has high sensitivity and specificity, and is therefore used to validate the gene expression study results obtained by microarray analysis. Other benefits of qRTPCR include real-time detection of gene amplification, detection of a very low level of gene expression and rapid analysis of gene expression [4]. However, to obtain the most accurate data possible, normalization and validation of qRTPCR results are needed. The most commonly used normalization technique uses an internal control reference gene [5]. The level of expression of gene of interest is determined with reference to a stably expressed reference gene.

The reference gene method compensates for variability introduced during all the stages of qRTPCR and RNA extraction. Reference genes are constitutively expressed genes that are responsible for basic cellular function maintenance required for cell survival. The expression of a reference gene is expected to remain unaffected by experimental conditions [6]. Additionally, the expression of a reference gene should not be affected by the phase and condition of bacterial growth. It is also recommended to choose a reference gene with a similar threshold cycle to the gene of interest. However, the reference gene should show the variability caused by technical or procedural error [7]. There are no universal reference genes, and they need to be validated for a given experimental condition [8]. The use of an inappropriate reference gene can lead to significantly different and even error results [9].

*Staphylococcus aureus* is one of the most important bacterial human pathogens responsible for causing wound infection, skin and soft tissue infection, urinary tract infection, and bloodstream infection. It is also one of the most common causes of difficult-to-treat biofilm-related infections, such as prosthetic joint infection and other implant infections [10]. Savirin and ticagrelor are known to have in vitro as well as in vivo antibiofilm activity against *S. aureus* [11,12].

Ticagrelor is a Food and Drug Administration-approved P2Y12 receptor inhibitor antiplatelet drug used to prevent thrombotic events in atherosclerotic cardiovascular disease patients [13]. In the in vitro experiment, ticagrelor showed dose-dependent antibiofilm activity by reducing *S. aureus* biofilm biomass by more than 85 % at 20 μg/mL concentration [12]. This drug inhibited *S. aureus* biofilm growth on subcutaneous disks in a pre-contaminated subcutaneous foreign body *S. aureus* infection mouse model [12]. Similarly, savirin is a low molecular weight, lipophilic synthetic novel molecule known to inhibit and treat biofilm-related *S. aureus* skin and subcutaneous tissue infection in mouse models [11]. This molecule disrupts the *agr* quorum-sensing system by inhibiting AgrA attachment to promoter regions, leading to the suppression of key virulence factors in *S. aureus* [11]. This inhibition makes *S. aureus* cells less competent to survive inside host cells, leading to their easy clearance [11]. Understanding the effect of these molecules on the expression of *S. aureus* genes can help to develop more effective treatment options for biofilm-related *S. aureus* infection, than currently available strategies.

Different reference genes have been evaluated and used to study gene expression in *S. aureus* [14,15]. This study tested the effect of savirin or ticagrelor treatment on the expression of sixteen candidate reference genes in a strong biofilm producer *S. aureus* isolate isolated from biofilm-related infection.

## 2. Materials and Methods

A methicillin-susceptible *S. aureus* (MSSA) clinical isolate isolated from a case of urinary tract infection at the pathology unit of the Townsville University Hospital was used in this study. The isolate was susceptible to cefazolin, cotrimoxazole, ciprofloxacin, gentamicin, flucloxacillin, rifampicin, and vancomycin. This strain was a strong biofilm producer because the optical density (OD) of reconstituted crystal violet stain retained by biofilm formed by this strain was greater than 4 × (average OD + 3 standard deviation of OD, of reconstituted crystal violet stain retained by negative control) [16]. The *S. aureus* strain stored at −80 °C was cultured in Luria–Bertani (LB) broth at 37 °C for 48 h. The bacterial broth was then discarded and the bacteria attached to the wall of the culture tube were scraped and subcultured in 0.5 % glucose containing LB (GLB) broth for further 24 h. This process induced ample biofilm production in the *S. aureus* strain used. The bacterial broth culture obtained thus was used for further experiments.

### 2.1. RNA Extraction

RNA was extracted from ticagrelor (12.5 µg/mL) treated, ticagrelor diluent (1% DMF) treated, savirin (10 µg/mL) treated, and savirin diluent (0.02% DMSO)-treated log phase *S. aureus* cultures using the Qiagen RNeasy mini kit following the user’s manual. These ticagrelor and savirin concentrations were used because they inhibited biofilm formation without inhibiting planktonic growth.

In short, 10^5^ CFU of *S. aureus* in 50 µL volume was added to 50 µL ticagrelor solution to make final volume 100 µL and final ticagrelor concentration 12.5 µg/mL in a 96 well flat bottom microtiter plate. For positive control, ticagrelor diluent was added in place of ticagrelor. Similarly, 10^5^ CFU of *S. aureus* in 50 µL volume was added to 50 µL savirin solution to achieve a final volume of 100 µL and a final savirin concentration of 10 µg/mL. For positive control, savirin diluent was added in place of savirin. The bacterial cultures were then incubated at 37 °C for 8 h. After the incubation, the bacterial broth was discarded and the biofilm formed was rinsed once with sterile distilled water to remove planktonic bacterial cells. The remaining bacterial deposit on the wall of microtiter plate wells was scraped to prepare bacterial suspension in distilled water for RNA extraction. RNA was extracted by using the Qiagen RNeasy mini kit—RNA clean-up to remove genomic DNA was performed by using spin column digestion followed by in-solution digestion. A Nanodrop 2000C spectrophotometer (Thermo Fisher Scientific, Waltham, MA, USA) was used to check the quality and quantity of RNA. To rule out any procedural error and to confirm the ticagrelor and savirin concentrations used inhibited biofilm formation, a parallel culture for each experiment was incubated at 37 °C for 24 h and biofilm assay was performed. The RNA extracted was stored at −20 °C until it was used for qRTPCR within 48 h. To monitor degradation, the quantity and quality of RNA was tested just after extraction and then just before use, with the help of Nanodrop. To rule out contamination during incubation of the treated bacterial suspensions, sterile ticagrelor and savirin diluents were used as negative control for the respective experiments.

### 2.2. Quantitative Real Time PCR (qRTPCR)

A Bio-Rad iTaq universal SYBR green one-step kit and the comparative *C*_t_ (ΔΔ*C*_t_) method were used to test the effect of ticagrelor or savirin treatment in the expression of sixteen different candidate reference genes [17]. These are commonly used reference genes in different gene expression studies in *S. aureus* (Table 1). The qRTPCR reaction volume (10 µL) contained 5 µL 2× iTaq universal SYBR green reaction mix, 0.125 µL iScript reverse transcriptase, 0.8 ng RNA template in 1 µL volume, 1 nM primer mix in 1 µL volume, and 2.9 µL nuclease-free water. The thermo-cycler parameters used were reverse transcription (50 °C, 10 min), polymerase activation and DNA denaturation (95 °C, 1 min), 40 cycles of denaturation at 95 °C for 10 s and annealing/extension + plate read at 60 °C for 30 s.

### 2.3. Stability Evaluation of the Candidate Reference Genes

Four different algorithms, delta Ct [25], normfinder [26], bestkeeper [27], and genorm [28] were used to determine the stability of potential reference genes. In the delta Ct method, the stability of genes is determined by calculating the average standard deviation of the relative expression of gene pairs within each sample [25]. Normfinder determines the stability by taking both intra-group and inter-group gene variability into account [26]. The bestkeeper algorithm uses the standard deviation (SD) of Ct values to rank the stability of potential reference genes [27]. Similarly, genorm determines the pairwise standard deviation of Ct values [28]. The overall comprehensive ranking of candidate reference genes was determined by calculating the geometric mean of ranking values of all the algorithms using https://www.heartcure.com.au/reffinder/ (accessed on 15 August 2022). The optimal number of reference genes required was determined using genorm [28]. For pairwise variation (V_n_/V_n+1_) < 0.15, n is the optimal number of reference genes required. Variances of reference genes are added successively and pairwise variance (V_n_/V_n+1_) is determined, where V_n+1_ is a variance sum and V_n_ is the variance of a prior sum.

## 3. Results

The specific PCR product was confirmed by a single melting-curve peak.

### 3.1. Stability Evaluation of Candidate Reference Genes for Savirin Treatment Experiment

Threshold cycle (Ct) values for the candidate reference genes for savirin treatment ranged from 18 to 33. The delta Ct algorithm reported that the most stable genes were *rpoD*, *spa*, and *gyrB* or *pyk*. Comparable results with delta Ct algorithm were reported by normfinder, where the most stable genes were *spa*, *pyk*, *rpoD*, and *gyrB*. Similarly, genorm reported that most stable genes were *fabD*, *proC*, and *rho*, while bestkeeper reported *fema*, *gapdH*, and *16s* as the most stable genes. In the comprehensive analysis of the findings of all the algorithms used, the most stable reference genes were *spa, rpoD*, and *pyk*. This result is comparable with that of delta Ct and normfinder algorithms. The least stable gene reported by delta Ct, genorm, and normfinder analyses was *fema*, while that for bestkeeper and comprehensive analyses was *pta* (Table 2).

### 3.2. Stability Evaluation of Candidate Reference Genes for Ticagrelor Treatment Experiment

Ct values for the candidate reference genes for the ticagrelor treatment experiment ranged from 13 to 30. The Delta Ct algorithm reported that the most stable genes were *gapdH, 16s*, and *gyrA*. Results comparable with the delta Ct algorithm were reported by genorm, with the most stable genes being *gapdH*, *gyrA*, and *spa*. Bestkeeper reported that the most stable genes were *gmk*, *rpoB* and *rpoD*. Similarly, normfinder reported that the most stable reference genes were *fema*, *proC* and *gyrB*. In comprehensive analysis, the most stable genes were *gapdH*, *gyrA*, and *gmk*. This result was comparable with that of delta Ct and genorm. The most unstable gene for all the algorithms including comprehensive analysis was *pta* (Table 3).

### 3.3. Optimal Number of Reference Genes Required

**Figure 1:**
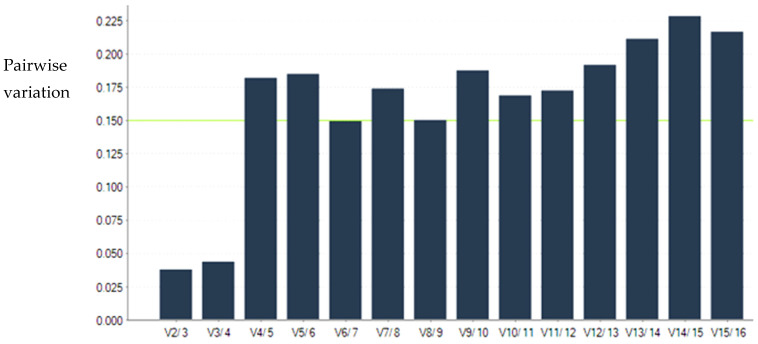
Determination of the optimal number of reference genes in savirin treatment experiment. V_2/3_ is less than 0.15, indicating the optimal number of reference genes required is two.

**Figure 2:**
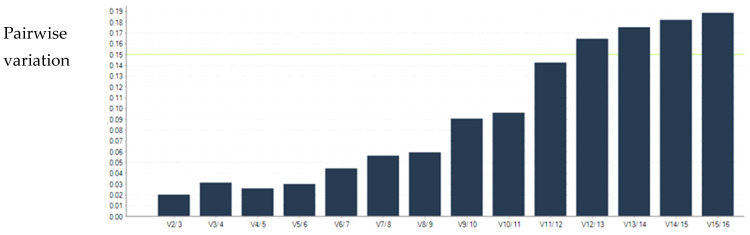
Determination of the optimal number of reference genes in ticagrelor treatment experiment. V_2/3_ is less than 0.15, indicating the optimal number of reference genes required is two.

For savirin and ticagrelor treatment experiments, V_2/3_ was less than 0.15. Therefore, the optimal number of reference genes required for both savirin and ticagrelor treatment experiments was two (Figure 1 and Figure 2).

## 4. Discussion

In this study, the effect of savirin or ticagrelor treatment on the expression of sixteen commonly used reference genes in *S. aureus* was tested. The concentrations of savirin (10 µg/mL) or ticagrelor (12.5 µg/mL) used for treatment were sufficient to prevent biofilm formation without significant planktonic growth inhibition. Four different algorithms—delta Ct [25], normfinder [26], bestkeeper [27], and genorm [28]—were used to determine the stability of the potential reference genes. However, none of these strategies are ideal, and therefore the consensus ranking of reference genes was determined by a comprehensive analysis of the results of all four methods used.

In the savirin treatment experiment, the comprehensive analysis of all the algorithms used reported that the most stable reference genes were *spa*, *rpoD*, and *pyk*, and the least stable gene was *pta*. Comparable results on the stability of reference genes were reported by delta Ct and normfinder, with the most stable genes in the former and latter methods being *rpoD*, *spa*, *gyrB*, or *pyk* and *spa*, *pyk*, *rpoD*, and *gyrB*, respectively. However, significantly different results were reported by genorm and bestkeeper, for which the most stable reference genes were *fabD*, *proC*, and *rho*, and *fema*, *gapdH*, and *16s*, respectively. Delta Ct, genorm, and normfinder analyses reported that the least stable reference gene was *fema*, while that for the bestkeeper method was *pta*.

Similarly, in the ticagrelor treatment experiment, the comprehensive analysis reported the most stable genes were *gapdH*, *gyrA*, and *gmk*, and the least stable gene was *pta*. Comparable results were reported by delta Ct and genorm algorithms, for which the most stable genes were *gapdH*, *16s*, and *gyrA*, and *gapdH*, *gyrA*, and *spa*, respectively. Similarly, bestkeeper reported that the most stable genes were *gmk*, *rpoB*, and *rpoD*, while normfinder reported that *fema*, *proC*, and *gyrB* were the most stable genes. The least stable gene reported by all the algorithms used was *pta*. Overall, there was a difference in the results of individual algorithms used, with some comparable results in both savirin and ticagrelor treatment experiments. There were some common appropriate reference genes reported by different algorithms for both savirin and ticagrelor treatment experiments. However, the overall difference in appropriate reference genes required to study the effect of savirin or ticagrelor treatment on *S. aureus* genes reported by this study further stresses the requirement for the validation of reference genes for each experimental condition.

Although a single reference gene has been used regularly in many studies, using two or more genes can provide more reliable results if small changes in gene expression are to be detected [28]. In this study, the optimal number of reference genes required for both savirin and ticagrelor treatment experiments was two.

Differences in the results of different methods used on the choice of reference genes with comparable results among some algorithms have already been reported [29,30,31]. These differences were expected, as different algorithms use different statistical methods. Therefore, the reference genes were finalized by combining the comprehensive ranking and the optimal number of reference genes required, as determined by genorm. The delta Ct method determines gene stability by calculating the average standard deviation of the relative expression (ΔCt) of gene pairs within each sample, assuming the genes are not co-regulated [25]. Similarly, the bestkeeper algorithm uses the standard deviation of Ct values to rank the stability of potential reference genes [27]. This method assumes that the input data are normally distributed. For the genorm algorithm, the input raw data do not need to be normally distributed. This algorithm uses the pairwise standard deviation of Ct values to determine the most stable reference gene [28]. The genorm algorithm determines stability based on the assumption that the ratio of the expression of the most reliable two reference genes must be the same in all test conditions. Genorm does not take inter-group variation into consideration and assumes that the genes are not biologically or experimentally co-regulated. Normfinder takes both intra-group and inter-group gene variability into account and prevents the risk of selecting co-regulated genes [26]. Overall, all these algorithms are based on the assumption that the expressions of candidate reference genes do not show systematic variation, and this may not be always true. Therefore, the use of an independent statistical tool that is free of this assumption and can assess the stability of the candidate reference genes independently, in combination with the algorithms, is recommended [32]. Overall, despite having some drawbacks, each algorithm can reliably determine an appropriate reference gene independently.

This is the first study to investigate the effect of savirin or ticagrelor treatment on the expression of several commonly used reference genes in *S. aureus*. The genes belonging to different bacterial biochemical pathways were chosen to minimize the inclusion of the co-regulated genes that might be affected by the same experimental conditions [15]. Savirin and ticagrelor inhibit *S. aureus* biofilm formation and can improve the treatment outcome of biofilm-related *S. aureus* infection [11,12].

A previous study that investigated the effect of 5 µg/mL savirin on the expression of *agr* gene used *16s* as a reference gene [11]. Additionally, the same study used microarray analysis and showed no effect of savirin (5 µg/mL) treatment on the expression of most of the candidate reference genes analyzed in this study [11]. The previous study incubated *S. aureus* with 5 µg/mL of savirin for 5 h in Tryptone Soy Broth with AIP1, while in this study, the bacteria were incubated with 10 µg/mL of savirin for 8 h in glucose containing Luria–Bertani broth. While the previous study did not report any antibacterial activity of savirin (5 µg/mL) against *S. aureus*, a higher concentration of savirin is known to be antibacterial [33]. Five µg/mL savirin has been reported to downregulate *agr* and some other *agr*-dependent genes [11]. Since the *agr* gene is responsible for *S. aureus* biofilm dispersal, 5 µg/mL savirin would have been expected to enhance biofilm formation in *S. aureus* [34]. Activation of the *agr* quorum-sensing system causes biofilm dispersal in *S. aureus* and disruption of this system promotes robust biofilm formation. However, this study reported no effect with 5 µg/mL savirin but reduced biofilm formation following treatment with 10 µg/mL. This difference in results between the previous and the current study might be due to the higher savirin (10 µg/mL) concentration used in this study, differences between the growth conditions, and the *S. aureus* strains used in the two studies.

To our knowledge, the effect of ticagrelor treatment on *S. aureus* potential reference genes has never been studied before. In this study, the *16s* gene, which has already been used as a reference gene in a savirin treatment experiment, was found to be stably expressed by the bestkeeper algorithm in the savirin treatment experiment and by all the algorithms used in the ticagrelor treatment experiment [11]. However, while the *16s* gene has high target copy numbers, its transcripts do not represent the overall *S. aureus* mRNA and therefore might not be an ideal internal control [15].

Among the most suitable reference genes for the savirin treatment experiment reported by comprehensive analysis, *spa* encodes Staphylococcal protein A, a *S. aureus* virulence factor that helps to survive against host immune responses [35]. This gene has been previously used as genetic marker for epidemiological and outbreak studies [36]. The gene *rpo* contributes to transcription and has been used as a reference gene in an experiment involving the treatment of *S. aureus* with manuka honey [15,23,37]. Similarly, the gene *pyk* is involved in glycolysis and has been found to be stably expressed when treated with rhodamine 6G or crystal violet or berberine [15]. Among the genes found to be most stably expressed by individual algorithms when treated with savirin, *fabD* helps in fatty acid biosynthesis and has been found to be stably expressed when treated with ethidium or berberine [15]. Similarly, *fema* gene is involved in peptidoglycan biosynthesis and has been used as a molecular marker for *S. aureus* identification [38].

Among the most suitable reference genes for the ticagrelor treatment experiment reported by comprehensive analysis, *gapdH* plays an important role in glycolysis and is used frequently as a reference gene in different experimental conditions, including in an experiment that studied the resistance mechanism of *S. aureus* against amoxicillin [22,39,40]. Gene *gyrA* takes part in replication and has also been found to be a suitable reference gene for ethidium treatment experiments [15]. Similarly, *gmk* is involved in nucleotide metabolism and is a suitable reference gene for gene expression study under photodynamic treatment, but it is the most unstable gene for crystal violet treatment experiment [15,40]. Among the suitable reference genes reported by individual algorithms, *proC* contributes to amino acid biosynthesis, and its expression was found to be least affected by the presence of rhodamine 6G, crystal violet, or berberine [15]. In this study, *tpiA* was the most unstable gene in both savirin and ticagrelor treatment experiments. This gene is involved in gluconeogenesis and has also been determined to be the most unstable gene in *S. aureus* crystal violet treatment experiment [15]. However, *tpiA* is an appropriate reference gene for ethidium treatment experiment [15]. Based on all of this information, it can be concluded that the reference genes are constitutively expressed genes responsible for basic cellular function maintenance. An appropriate reference gene should be confirmed for each experimental condition.

This study used only one *S. aureus* strain, and therefore the results cannot be generalized. More studies using different *S. aureus* strains, including reference strains, are recommended. The expression levels of reference genes might differ among different *S. aureus* strains, and even in the same strain with changes in growth conditions. Therefore, we do not recommend direct use of the most stable reference genes reported in this study without prior re-validation.

On the basis of consensus ranking and the optimal number of reference genes reported by genorm, *spa* and *rpoD* were the most appropriate reference genes for savirin treatment experiment, while *gapdH* and *gyrA* were most suitable for ticagrelor treatment experiment. This study provides a foundation for gene expression studies using savirin or ticagrelor treatment in *S. aureus*.

## Figures and Tables

**Table 1:** Primers for candidate reference genes used in qRTPCR.

Genes	Forward Primer	Reverse Primer	References
*glyA*	CTACAAACTCACAGCCAC	GTATCGGAAGCGGTTATG	[15]
*gmk*	CCATCTGGAGTAGGTAAAGG	CTACGCCATCAACTTCAC	
*gyrA*	GTGTTATCGTTGCTCGTG	CGGTGTCATACCTTGTTC	
*proC*	GGCAGGTATTCCGATTGA	CCAGTAACAGAGTGTCCAAC	
*pyk*	GCATCTGTACTCTTACGTCC	GGTGACTCCAAGTGAAGA	
*fabD*	CCTTTAGCAGTATCTGGACC	GAAACTTAGCATCACGCC	
*recF*	AGTTATAGACACGGCACG	GCGTCGTCTTATTTGAGG	
*rho*	GGAAGATACGACGTTCAGAC	GAAGCGGGTGGAAGTTTA	
*rpoD*	CACGAGTGATTGCTTGTC	GATACGTAGGTCGTGGTATG	
*gyrB*	GGTGCTGGGCAAATACAAGT	TGGGATACCACGTCCGTTAT	[18]
*spa*	AGCACCAAAAGAGGAAGACAA	GTTTAACGACATGTACTCCGT	[19]
*fema*	TGCCTTTACAGATAGCATGCCA	AGTAAGTAAGCAAGCTGCAATGACC	[20]
*pta*	AGAAGCAATCATTGATGGCGA	ACCTGGCGCTTTTTTCTCAG	[21]
*gapdH*	TGACACTATGCAAGGTCGTTTCAC	TCAGAACCGTCTAACTCTTGGTGG	[22]
*rpoB*	CAGCTGACGAAGAAGATAGCTATGT	ACTTCATCATCCATGAAACGACCAT	[23]
*16s*	AGAGATAGAGCCTTCCCCTT	TTAACCCAACATCTCACGACA	[24]

**Table 2:** Stability of different candidate reference genes when treated with savarin, as reported by different algorithms.

	Delta Ct		Genorm		Bestkeeper			Normfinder		Comprehensive	
Rank	Genes	Stability Value	Genes	Stability Value	Genes	Mean Ct Value	SD	Genes	Stability Value	Genes	Geometric Mean
1	*rpoD*	1.62	*fabD*	0.028	*fema*	26.56	0.09	*spa*	0.060	*spa*	3.35
2	*spa*	1.62	*proC*	0.028	*gapdH*	20.08	0.1	*pyk*	0.067	*rpoD*	3.72
3	*gyrB*	1.63	*rho*	0.085	*16s*	18.06	0.75	*rpoD*	0.067	*pyk*	4.74
4	*pyk*	1.63	*gyrA*	0.130	*glyA*	31.20	1.17	*gyrB*	0.199	*gyrB*	5.18
5	*rpoB*	1.73	*rpoB*	0.441	*gmk*	25.74	1.44	*rpoB*	0.803	*rpoB*	6.09
6	*recF*	1.81	*gyrB*	0.664	*recF*	26.81	1.56	*recF*	0.847	*proC*	6.42
7	*gmk*	1.88	*spa*	0.772	*pyk*	27.01	2.03	*gmk*	1.052	*recF*	6.82
8	*gyrA*	2.05	*rpoD*	0.927	*rpoD*	28.93	2.13	*glyA*	1.491	*fabD*	6.82
9	*glyA*	2.09	*pyk*	1.021	*spa*	25.75	2.48	*gyrA*	1.705	*gmk*	7.21
10	*rho*	2.10	*recF*	1.191	*gyrB*	30.26	2.56	*rho*	1.815	*gyrA*	7.67
11	*proC*	2.16	*gmk*	1.312	*rpoB*	22.11	2.83	*proC*	1.925	*glyA*	7.67
12	*fabD*	2.19	*glyA*	1.438	*gyrA*	29.02	3.38	*fabD*	1.956	*rho*	7.90
13	*16s*	2.48	*16s*	1.600	*rho*	32.20	3.45	*16s*	2.150	*fema*	8.00
14	*gapdH*	3.21	*pta*	1.793	*proC*	30.87	3.52	*gapdH*	3.143	*gapdH*	8.76
15	*pta*	3.40	*gapdH*	2.010	*fabD*	31.20	3.54	*pta*	3.373	*16s*	9.01
16	*fema*	3.46	*fema*	2.192	*pta*	29.10	4.46	*fema*	3.434	*pta*	14.98

**Table 3:** Stability of different candidate reference genes when treated with ticagrelor as reported by different algorithms.

	Delta Ct		Genorm		Bestkeeper			Normfinder		Comprehensive	
Rank	Genes	Stability Value	Genes	Stability Value	Genes	Mean Ct Value	SD	Genes	Stability Value	Genes	Geometric Mean
1	*gapdH*	1.09	*gapdH*	0.035	*gmk*	22.89	0.05	*fema*	0.120	*gapdH*	2.34
2	*16s*	1.09	*gyrA*	0.035	*rpoB*	13.23	0.08	*proC*	0.141	*gyrA*	3.08
3	*gyrA*	1.09	*spa*	0.052	*rpoD*	26.66	0.08	*gyrB*	0.270	*gmk*	4.21
4	*spa*	1.10	*rpoB*	0.088	*spa*	26.88	0.14	*16s*	0.404	*spa*	4.28
5	*proC*	1.12	*gmk*	0.105	*gyrA*	27.11	0.16	*gapdH*	0.522	*16s*	4.43
6	*rpoB*	1.14	*16s*	0.130	*gapdH*	21.08	0.19	*gyrA*	0.568	*rpoB*	4.43
7	*gmk*	1.16	*rpoD*	0.181	*recF*	24.25	0.21	*spa*	0.622	*fema*	5.32
8	*fema*	1.24	*recF*	0.248	*16s*	18.42	0.25	*rpoB*	0.726	*proC*	5.33
9	*rpoD*	1.27	*proC*	0.312	*proC*	29.98	0.45	*gmk*	0.768	*rpoD*	6.59
10	*gyrB*	1.34	*fema*	0.430	*fema*	27.04	0.75	*rpoD*	0.975	*gyrB*	7.76
11	*recF*	1.42	*gyrB*	0.544	*gyrB*	29.07	0.92	*recF*	1.183	*recF*	9.07
12	*rho*	1.74	*rho*	0.738	*pyk*	25.91	1.17	*rho*	1.209	*rho*	12.24
13	*fabD*	2.22	*pyk*	0.954	*rho*	27.67	1.46	*fabD*	2.003	*fabD*	13.49
14	*glyA*	2.58	*fabD*	1.168	*fabD*	24.58	1.97	*glyA*	2.493	*pyk*	13.69
15	*pyk*	2.69	*glyA*	1.376	*glyA*	24.01	2.29	*pyk*	2.667	*glyA*	14.49
16	*pta*	3.01	*pta*	1.581	*pta*	24.05	2.62	*pta*	2.997	*pta*	16.00

## Data Availability

All the relevant data related to this study are presented in the manuscript.
